# Pattern of Antibiotic Prescription among Dentists in Iran

**Published:** 2007-04-01

**Authors:** Shahla Kakoei, Maryam Raoof, Fahimeh Baghaei, Shahrzad Adhami

**Affiliations:** 1 *Department of Oral Medicine, Dental School, Kerman University of Medical Sciences, Kerman, Iran*; 2 *Department of Endodontics, Dental School, Kerman University of Medical Sciences, Kerman, Iran*; 3 *Department of Oral Pathology, Dental School, Kerman University of Medical Sciences, Kerman, Iran*

**Keywords:** Antibiotic, Prescription

## Abstract

**INTRODUCTION:** This study examines the antibiotic prescription in dentists participated in 9^th^ Congress of Iranian Association of Endodontists in Esfahan/2006.

**MATERIALS AND METHODS:** A questionnaire for this cross sectional study was designed for evaluating the patterns of antibiotic prescription. It included some demographic information, clinical signs, and conditions in need for antibiotic and prophylactic prescription and their choices. Data was analyzed using Pearson’s Chi-square test.

**RESULTS:** High percentage of responders prescribe antibiotic for fever (78.2%) and diffuse swelling (85.1%). However, some situations such as acute pulpitis, chronic periapical lesions and marginal gingivitis were irrationally prescribed.

**CONCLUSION:** This study showed a fairly good pattern of antibiotic prescription but it was far from ideal.

## INTRODUCTION

There are different microorganisms exist in oral cavity. In some conditions, they can enter blood via disintegrated epithelium of oral cavity causing many complications such as bacterial endocarditis. An appropriate use of antibiotics can shorten the duration of bacterial infection and decrease complications such as infection spread to adjacent anatomical spaces or systemic involvement. However, antibiotics are prescribed in different clinical situations (1).

Antibiotics are common drugs used either for prophylaxis or as a part of the management of orofacial infections (2). Antimicrobial resistance is a rapid increasing problem due to irrational use of antibiotics (3). One of the contributor factors is inappropriate use of antibiotics in veterinary medicine, agriculture, medicine, and probably dentistry, but dentistry’s contribution in antimicrobial resistance is unknown (1). This problem has emphasized the need for rationalization of antibiotic use in treatment of infections (4).

The survey of past studies revealed appreciable gap in knowledge with respect to rational drug use. For this reason, these researches suggest an urgent need for review of current policies and systems with the view of enhancing the drug use practices of health providers (2-4). These findings were in agreement with previous results in Iran (5-6).

Although the findings of a recent study have shown that general practitioners prescribe therapeutic and prophylactic antibiotics, inappropriately (4). To our best knowledge, data of prescribing habits of dental practitioners is scarce. Therefore, this study was carried out to determine dentists’ current knowledge and behavior regarding antibiotic prescription in Iran.

## MATERIALS AND METHODS

A questionnaire for this cross-sectional study was designed to investigate dental practitioners' behavior of prescribing antibiotics therapeutically and prophylactically, the both.

After evaluating the validity and reliability of the questionnaire, it was given to all participants of the 9th Congress of Iranian Association of Endodontists which was hold in Esfahan, August 2006.

**Table 1:** Demographic and professional chara-cteristics of participating dentists

**Variable**	
**Gender:**	
Male	68 (63.6%)
Female	39 (36.4%)
**Age**	24-70
**Year of education**	0-38
**Post graduation course(s):**	
Yes	40
No	66

Filled questionnaires were collected from participants. At first, the questionnaire information was sought on the gender, age, being general or specialized dentist, and record of service.

Participants were asked to give answers to some clinical signs and conditions required antibiotics and their choice of medicine. Based on other studies, the following clinical signs were questioned: fever, diffuse swelling, trismus, acute pulpitis, localized periapical abscess, chronic periapical lesion, chronic marginal gingivitis, chronic periodontitis, tooth reimplantation, dry socket, before and after root canal therapy, before and after extraction, before and after dental surgery (1-4).

Respondents were required to provide information on the preferred antibiotics and their choice of alternatives for patients who are allergic to penicillin.

The next part of the questionnaire covered the patients who needed prophylactic antibiotic. Based on the recommendations of the American Heart Association, the following dental procedures were questioned: periodontal probing, scaling, supra-gingival and sub-gingival restorations, matrix band placement, tooth extraction, block and PDL injections, fluoride therapy, taking radiography, molding and root canal therapy.

The collected data were computerized and processed using SPSS soft ware (version 13), and Pearson's Chi-square test was used for statistical analysis.

## RESULTS

Totally, one hundred and thirteen participants filled the questionnaires. Of these, 62.3% were general practitioners and remains (37.7%) as dental specialists.

**Table 2.** Systemic antibiotic prescription for different situation

**Antibiotic prescription Situations**	**Always, almost, sometimes **	**Rarely, never**
Fever	79(78.2)^a^	22(21.8)
Acute pulpitis	27(26.7)	74(73.3)
Local periapical abscess	45(44.6)	56(55.4)
Chronic periapical abscess	28(27.7)	73(72.3)
Chronic marginal gingivitis	14(13.9)	87(86.1)
Chronic periodontitis	21(20.8)	80(79.2)
Unexplained trismus	37(36.6)	64(63.4)
Diffuse swelling	86(85.1)	15(14.9)
Tooth reimplantation	61(60.4)	40(39.6)
Dry socket	25(24.8)	76(75.2)
Before endodontic therapy	52(51.5)	49(48.5)
After endodontic therapy	24(23.8)	77(76.2)
Before tooth extraction	24(23.8)	77(76.2)
After tooth extraction	36(35.6)	65(64.4)
Before tooth surgery	39(38.6)	62(61.4)
After tooth surgery	65(64.4)	36(35.6)

***a:*** Number (percent

Demographic and professional characteristics of respondents are shown in Table 1. No significant difference was identified between the correct antibiotic prescription regarding demographic data including age and gender. Only level of education demonstrated some statistically significant differences including positive answer to antibiotic prescription before tooth extraction (31% dental practitioners and 13.2% specialists), after tooth extraction (18.4% dental practitioners and 48.3% specialists), after tooth surgery (94%dental practitioners and 36% specialists), erythromycin prescription after root canal therapy in the case of allergy to penicillin (79% dental practitioners and 43% specialists), but in this situation dental specialists prefer clindamycin (64.9%).

The clinical signs for which the respondents would prescribe antibiotics are shown in Table 2. The most abuse of antibiotics was related to chronic periodontitis (79.2%). Nevertheless, a noticeably higher correct response rate was recorded in the cases of diffuse swelling (85.1%) and fevers (78.2%). The lists of antibiotics that have been chosen by the respondents for specific oral and dental conditions in patients with or without allergy to penicillin are shown in Table 3 and Table 4.

**Table 3:** Prescription of antibiotic after RCT by practitioners

**Antibiotic prescription ** **Antibiotic**	**Always Almost, Sometimes**	**Rarely Never**
Amoxicillin	91 (90.1)^ a^	10 (9.9)
Metronidazole	70 (69.3)	31 (30.7)
Co-amoxiclave	15 (14.9)	86 (85.1)
Ampicillin	17 (16.8)	84 (83.2)
Penicillin	51 (50.5)	50 (49.5)
Erythromycin	16 (15.8)	85 (84.2)

***a:*** Number (percent

**Table 4:** Antibiotic prescription in patients with allergy to penicillin

**Antibiotic prescription ** **Antibiotic**	**Always Almost Sometimes**	**Rarely Never**
Erythromycin	66 (66)^ a^	34 (34)
Metronidazole	27 (27)	73 (73)
Azithromycin	12 (12)	88 (88)
Amoxicillin	12 (12)	88 (88)
Co-amoxiclave	5 (5)	95 (95)
Clindamycin	44 (44)	56 (56)
Cephalexin	22 (22)	78 (78)
Tetracyclin	11 (11)	89 (89)

***a:*** Number (percent

Amongst all, amoxicillin was the most preferred antibiotic in patients who were not allergic to Penicillin followed by metronidazole. In patients who are allergic to penicillin, erythromycin and clindamycin were the most prescribed alternatives. Regarding prophylactic antibiotic prescription for patients with the risk of bacterial endocarditis data is shown in Table 5. In some conditions such as periodontal probing and nerve block injection, the respondents didn’t show enough awareness.

## DISCUSSION

The findings of this study might reflect a fairly good pattern of antibiotic prescription but far from ideal. Results of many studies confirm it indicating that antibiotics were not always prescribed on a scientific basis (2,5,7-9).

A large percentage of responders would prescribe antibiotics for cases in which oral infection is accompanied by fever and evidence of diffuse swelling. Therefore, practitioners’ knowledge is well on these items. Interestingly, the data on the management of unexplained trismus, chronic periodontitis and tooth replantation showed under usage of antibiotics. About 20% would prescribe antibiotics irrationally for acute pulpitis, chronic periapical lesions, chronic marginal gingivitis, dry socket, before and after root canal therapy, before and after extraction, and before the third molar surgery in a healthy person. It is predictable that heavy work load in some of the dental centers and consequently the lack of enough time to evaluate the patient’s actual need can cause this potential abuse of the antibiotics. In other words, this abuse may be due to lack of information about the indications and side effects of antibiotics.

**Table 5:** Different procedures that practitioners prescribe prophylactic antibiotic

**Antibiotic prescription Variable situation**	**Always Almost Sometime**	**Rarely Never**
Scaling	88(83)^a^	18(17)
Supra-gingival restoration	19(17.9)	87(82.1)
Sub-gingival restoration	84(79.2)	22(20.8)
Fluoride therapy	8(7.5)	98(92.5)
Matrix band placement	77(72.6)	29(27.4)
Probing	63(59.4)	43(40.6)
RCT with proper WL	51(48.1)	55(51.9)
Taking radiography	4(3.8)	102(96.2)
Tooth extraction	97(91.5)	9(8.5)
Reimplantation	87(82.1)	19(17.9)
Impression	20(18.9)	86(81.1)
Falling deciduous tooth	12(11.4)	93(88.6)
Block injection	56(53.3)	49(46.7)
Intra-ligamentary injection	82(78.1)	23(21.9)
Infiltration injection	42(40)	63(60)

***a:*** Number (percent

Meanwhile, sometimes the practitioners would prescribe antibiotics for unscientific reasons like patients’ expectation to satisfy them.

The proportion of practitioners who would routinely prescribe antibiotics for specific conditions varies a great deal among the presenting diseases. For example, about half of the practitioners were giving antibiotics incorrectly for chronic periapical abscess with a sinus tract, after root canal therapy, and third molar surgery. In majority of localized or diffuse infections, removal of the etiology and/or providing drainage would usually lead to a complete resolution of the problem (2).

The incidence of infective osteitis after the minor oral surgery due to periradicular or third molar surgeries has drastically reduced with improvement of aseptic techniques and better instrumentation. Therefore, antibiotic usage is not routinely necessary in these situations (10-11). Ramezanian stated that antibiotic therapy before surgery does not seem necessary, if the non-traumatic surgery will be in an aseptic condition (12). Comparing the advantages and disadvantages of antibiotics giving in these situations, it is concluded that irrational use of antibiotics will lead to adverse effects, anti-microbial resistance and economic determent.

In this study, the most frequently prescribed systemic antibiotics in dental infections were amoxicillin and penicillin, respectively. Based on the literatures (13-14), penicillin VK remains the antibiotic of choice for treatment of endodontic infections because of its efficacy and low toxicity. Many investigations showed the most prescribed antibiotic was amoxicillin which is in the line with the current study (2,5,15-17). These results indicate that the use of penicillin is gradually being reduced as a study has shown that the main microorganisms isolated from dental abscess are complex mixture of facultative and anaerobic bacteria, many of which are penicillin resistant (2).

There were wide variations of antibiotic regimens use. This result was similar to those of Kandemir and Ergul (18), Gatewood *et al.* (19), and Seltzer and Naidorf (20).

Regarding allergy to one of penicillins, patient should be considered allergic to all penicillins and probably to cephalosporins as well (23). For these patients erythromycin is the common-est alternative followed by clindamycin, metronidazole, and cephalexin, respectively. This result agrees previous studies (2,6). Erythromycin overuse may be due to a) its recommendation in some previous literatures, b) being a common antibiotic, and c) it’s low price. Clindamycin is recommended for patients who are allergic to penicillin (21). Its association with acute pseudomembranous colitis should be considered as an important limitation (22).

All periodontal treatment procedures (including probing) require antibiotic prophylaxis in patients at risk for infective endocarditis (24). Endocarditic prophylaxis is not recommended for non-intraligamentary local anesthesia, rubber dam placement, or taking radiographs (21). Pattern of antibiotic prescription regarding this point was fairly good in the current study. About more than 80% of responders would prefer prophylactic antibiotic exactly before scaling, subgingival restoration, matrix band placement, tooth extraction and PDL injection for patients who are at risk of infective endocarditis. Only few percents of practitioners prescribe antibiotic irrationally before supragingival restorations, fluoride therapy, taking radiography and molding. In the instance of probing, block injection and root canal therapy with a correct working length (WL), the practitioners information were not good at all and about half of the responders’ answers were incorrect in this respect.

We expected that higher education would result in better antibiotic prescription pattern, but in this study it was not applicable to most of the questions. The only significant difference was observed between the level of education and antibiotic prescription pattern before and after tooth extraction and after third molar surgery. In the case of a patient who needs antibiotic after root canal therapy and is allergic to penicillin, specialists’ knowledge is statistically higher than general practitioners.

The limitation of the present study, including the small number of participants should be considered and indicating the need for further work. Although more researches are needed, accumulating evidence suggests that dental practitioners’ knowledge about the use of antibiotics is far from ideal. Rational prescribing based on a thorough knowledge is an important objective. Some ways which might play the major roles to achieve this issue are dentists participating in the regularly continuing dental education courses in the field of antibiotics usage, effective communication between microbiologists and practitioners, re-evaluation and standardizing the teaching in the use of antibiotics. In addition, greater emphasis on the training of clinical students about antimicrobial agents could be beneficial in this crucial part of their work.

## CONCLUSION

The results of the present study have demonstrated a lack of uniformity in the rational use of antibiotic among dental practitioners in Iran.
